# Role of cachexia in advanced non-small cell lung cancer patients treated with EGFR-TKIS

**DOI:** 10.1186/s12885-026-15773-1

**Published:** 2026-04-02

**Authors:** Jenny G. Turcott, Cittim B. Palomares-Palomares, Eduardo Rios-Garcia, Daniela Cardenas-Fernandez, Diego A. Diaz-Garcia, Salvador Gutiérrez Torres, Andrés F. Cardona, Oscar Arrieta

**Affiliations:** 1 Thoracic Oncology Unit, Instituto Nacional de Cancerología (INCan), Av. San Fernando #22, Sección XVI, Tlalpan, 14080 Mexico City, Mexico; 2 Facultad de Ciencias Administrativas y Sociales, Universidad Autónoma de Baja California, Ensenada, Mexico; 3 Radio-oncology Department, Instituto Nacional de Cancerología (INCan), Mexico City, 14080 Mexico; 4 Thoracic Oncology Unit and Direction of Research and Education, Luis Carlos Sarmiento Angulo Cancer Treatment and Research Center, Bogotá, Colombia

**Keywords:** EGFR, Tyrosine kinase inhibitors, Toxicity, Cachexia, NSCLC

## Abstract

**Introduction:**

Cachexia is a multifactorial syndrome associated with impaired physiologic reserve and adverse outcomes in advanced NSCLC. Its relationship with early toxicity and survival during EGFR-tyrosine kinase inhibitor (EGFR-TKI) therapy remains insufficiently characterized. We evaluated the impact of baseline cachexia on toxicity and clinical outcomes in patients with EGFR-mutated stage III–IV NSCLC treated with EGFR-TKIs.

**Patients and methods:**

In this retrospective cohort, 247 consecutive patients treated with EGFR-TKIs were included. Cachexia was defined using the Fearon international consensus criteria. Hematologic and selected non-hematologic adverse events were recorded during the first four months of therapy. Associations with hematologic toxicity were assessed using logistic regression, and progression-free survival (PFS) and overall survival (OS) were evaluated using Kaplan–Meier methods and Cox regression.

**Results:**

Baseline cachexia was present in 91 patients (37%). Cachectic patients more frequently had KPS <90 (44% vs 27%) and liver metastases (19% vs 5.8%). Overall hematologic toxicity was higher among cachectic versus non-cachectic patients (85.7% vs 69.9%), driven mainly by anemia (40% vs 21%) and lymphopenia (74% vs 56%). Cachexia independently predicted hematologic toxicity (adjusted OR 2.26, 95% CI 1.15–4.68) and worse OS (adjusted HR 1.65, 95% CI 1.07–2.57); median OS was 37.22 months in cachectic patients versus 45.80 months in non-cachectic patients.

**Conclusions:**

Baseline cachexia was associated with a higher burden of early hematologic toxicity and independently worse OS in EGFR-mutated advanced NSCLC treated with EGFR-TKIs. Routine baseline identification may help prioritize early supportive care and closer monitoring during the initial months of therapy.

**Supplementary Information:**

The online version contains supplementary material available at 10.1186/s12885-026-15773-1.

## Introduction

Cancer-associated cachexia is a major concern when indicating oncological treatment because of its contribution to morbidity and mortality [1]. Epidermal growth factor receptor tyrosine kinase inhibitor (EGFR-TKI)-based treatments involve gastrointestinal toxicity, necessitating adequate management to minimize it and avoiding nutritional status deterioration, dose-limiting toxicity (DLT), and treatment discontinuation [2]. Diarrhea is a frequent adverse effect, with a prevalence ranging from 18% to 95%. Chronic diarrhea can cause significant nutrient loss and malabsorption, resulting in severe malnutrition and, ultimately, cachexia [3].

Understanding how diarrhea-induced malnutrition can lead to cachexia and/or sarcopenia is crucial for prioritizing care during treatment. Sarcopenia, defined as the wasting of skeletal muscle mass, is the main feature of cancer cachexia and is associated with fatigue, increased catabolism, poor tolerance to treatment, and poor prognosis [4]. In patients with cancer, computed tomography (CT) is routinely used to assess tumors and monitor treatment responses. Imaging of the third lumbar vertebra has been employed to evaluate the distribution of adipose tissue and skeletal muscle areas, significantly correlating with the overall body muscle mass. This method is considered the gold standard for detecting low muscle mass among cancer patients, and a cutoff point to determine sarcopenia in patients with Non-Small Cell Lung Cancer (NSCLC) has already been established [5].

In addition to sarcopenia, cachexia can present with or without fat mass loss and may involve decreased muscle strength, fatigue, anorexia, increased inflammatory markers (C-reactive protein [CRP], Interleukin-6 [IL-6]), anemia (hemoglobin < 12 g/dL), and/or low serum albumin (< 3.2 g/dL) [6]. The potential influence of cachexia and/or sarcopenia on the effectiveness of EGFR-TKIs and their potential side effects are not fully understood [7]. However, cachexia may affect treatment outcomes by exacerbating toxicity profiles. Patients with cachexia are more vulnerable to the adverse effects of EGFR-TKIs, such as gastrointestinal toxicity, including severe diarrhea. This toxicity not only compromises nutritional status, but also increases the risk of treatment interruptions and dose reductions, which can directly impact treatment efficacy and patient survival.

The objective of this study was to explore the role of cachexia in TKI-associated toxicity, and its impact in clinical outcomes

## Materials and methods

### Data collection

This retrospective cohort study included consecutive patients at the Thoracic Oncology Unit of Instituto Nacional de Cancerologia (INCan) in Mexico City, between February 2015 and May 2022. The need for informed consent was waived because the study was conducted retrospectively. Eligibility required diagnosis of advanced NSCLC, and the presence of a pathogenic activating EGFR mutation confirmed by PCR or next-generation sequencing, with pre-treatment CT scans available that were readable at the L3 vertebra. Exclusion criteria were set for patients lacking readable L3 CT scans or adequate toxicity monitoring. The study was approved by the Comité de Ética en Investigación of the Instituto Nacional de Cancerología (INCan), Mexico City (approval No. 2024/068), and was conducted in accordance with the Declaration of Helsinki. The requirement for informed consent was waived due to the study’s retrospective design.

Data were obtained from the institution’s electronic medical records by the thoracic oncology team and verified by the research and nutrition teams. These information included sociodemographic and disease characteristics, hematological (anemia, thrombocytopenia, leukopenia, lymphopenia, and neutropenia) non-hematologic toxicity (constipation, fatigue, vomiting, nausea, anorexia, mucositis, xerosis, neuropathy, diarrhea, rash, paronychia, and hypoalbuminemia), weight changes, sarcopenia, body composition variables. Toxicity was considered at any grade according to the Common Terminology Criteria for Adverse Events (CTCAE) version 5.0, for the first four months of EGFR-TKI-based treatment, and the response assessment after the TKI-based treatment was reported. Additionally, overall hematologic toxicity and EGFR-TKI treatment changes due to toxicity (dose reduction, temporary interruption, or permanent discontinuation) were collected. Dose-limiting toxicity (DLT) was defined as any toxicity that prompted a documented dose reduction or temporary interruption of the EGFR-TKI.

### Cachexia evaluation

Cachexia was assessed at baseline and defined using the Fearon et al international consensus criteria: >5% weight loss over the previous 6 months, or >2% weight loss in patients with BMI <20 kg/m^2^ or with sarcopenia [8]. Body composition was assessed with the third lumbar vertebra (L3), extracted by a specialist operator, and contrasted with a supervising operator, using axial CT sections to estimate the demarcation of muscle and adipose tissue area measurement (cm^2^) using the Hounsfield unit (HU) threshold. Pre-specific HU threshold was used to determine skeletal muscle index (SMI), visceral adipose tissue (VAT), subcutaneous adipose tissue (SAT), and intramuscular adipose tissue (IAT) and they were divided by height squared to establish indices (VATI, SATI, and IATI). Sarcopenia was defined as an SMI cutoff point of ≤52.4 cm^2^/m^2^ for men and ≤38.5 cm^2^/m^2^ for women according to previous literature at the start of treatment [4].

### Statistical analysis

The participants were classified according to the identification of cachexia. Quantitative variables are described as median and 25-75th percentile. Categorical variables are described as frequencies and percentages. Differences between groups were analyzed using the Mann-Whitney U test for numerical variables and chi-square test for categorical variables. For categorical variables with less than five subjects in some categories, Fisher´s exact test was used.

The odds ratio (OR) was calculated in terms of hematological toxicity and independent covariates. including cachexia were analyzed using a logistic regression model. OS was calculated from EGFR-TKIs initiation to death due to any cause and PFS from the first TKI administration to recorded progression according to RECIST 1.1. Kaplan-Meier and log-rank tests were performed. Covariates included in the multivariate Cox Hazard model to explain survival were selected with a *p*-value of <0.05. Post-hoc power analysis confirmed that the observed sample size (n=247, with 91 cachectic patients) provided >80% power to detect hazard ratios ≥1.6 for overall survival at a two-sided α=0.05. Data were analyzed using SPSS software package version 26 (IBM Corp) and RStudio 2023.03.0+ 386 “Cherry Blossom” for Windows.

## Results

Among 356 patients screened, 247 patients were included in the analysis (Figure S1), Overall, 91 (37%; 95% CI, 31–43%) were classified as cachectic and 156 (63%; 95% CI, 57–69%) as non-cachectic. Across our sample, 163 patients were female (66%) and 84 were male (34%), with a similar sex distribution between cachectic and non-cachectic groups (female: 63% vs 68%; *p=*0.477). Median age was 64 years (IQR, 55–72) and did not differ by cachexia status (*p=*0.693). Baseline clinicopathologic features were largely balanced across groups, including *EGFR* subtype (*p=*0.163), tobacco exposure (*p=*0.316), ECOG performance status (*p=*0.195), clinical stage (*p=*0.428), brain metastases (*p*>0.999), treatment line (*p=*0.712), and TKI type (*p=*0.404) (Table 1). However, cachectic patients had significantly worse functional status by Karnofsky score (KPS <90: 44% vs 27%; *p=*0.009). Cachectic patients also had a higher frequency of liver metastases (19% vs 5.8%; *p=*0.003). Consistent with the cachexia phenotype, cachectic patients had lower body weight (median 58 vs 62 kg; *p<*0.001) and BMI (median 22.8 vs 25.4 kg/m^2^; *p<*0.001), and a higher prevalence of sarcopenia (55% vs 35%; *p=*0.003). Body composition measures indicated lower adiposity among cachectic patients, including lower visceral adipose tissue (VAT median 95 vs 120; *p=*0.006) and subcutaneous adipose tissue (SAT median 119 vs 175; *p<*0.001), with similarly lower VATI and SATI (*p=*0.002 and *p<*0.001, respectively).

**Table 1:** Clinical characteristics according to Cachexia status

Variable	Overall *N* = 247^1^	No *N* = 156^1^	Yes *N* = 91^1^	*p-value* ^*2*^
Sex				0.477
Female	163/247 (66%)	106/156 (68%)	57/91 (63%)	
Male	84/247 (34%)	50/156 (32%)	34/91 (37%)	
Age (years)	64 (55–72)	64 (56–73)	65 (55–72)	0.693
Age (≥60)				0.638
< 59	89/247 (36%)	54/156 (35%)	35/91 (38%)	
≥ 60	158/247 (64%)	102/156 (65%)	56/91 (62%)	
Smoking status				0.316
Negative	157/247 (64%)	95/156 (61%)	62/91 (68%)	
Positive	90/247 (36%)	61/156 (39%)	29/91 (32%)	
WSE				0.946
No	169/247 (68%)	106/156 (68%)	63/91 (69%)	
Yes	78/247 (32%)	50/156 (32%)	28/91 (31%)	
Asbestos exposure				0.297
No	223/247 (90%)	138/156 (88%)	85/91 (93%)	
Yes	24/247 (9.7%)	18/156 (12%)	6/91 (6.6%)	
KPS				**0.009**
< 89	82/247 (33%)	42/156 (27%)	40/91 (44%)	
≥ 90	165/247 (67%)	114/156 (73%)	51/91 (56%)	
ECOG PS				0.195
0–1	214/247 (87%)	139/156 (89%)	75/91 (82%)	
≥ 2	33/247 (13%)	17/156 (11%)	16/91 (18%)	
Clinical Stage				0.428
III	10/247 (4.0%)	8/156 (5.1%)	2/91 (2.2%)	
IV	237/247 (96%)	148/156 (95%)	89/91 (98%)	
Sarcopenia				**0.003**
No	143/247 (58%)	102/156 (65%)	41/91 (45%)	
Yes	104/247 (42%)	54/156 (35%)	50/91 (55%)	
*EGFR* mutation type				0.163
*EGFR*^*del19*^	162/247 (66%)	105/156 (67%)	57/91 (63%)	
*EGFR*^*L858R*^	76/247 (31%)	48/156 (31%)	28/91 (31%)	
Uncommon mutations	9/247 (3.6%)	3/156 (1.9%)	6/91 (6.6%)	
Brain Mets				>0.999
No	146/247 (59%)	92/156 (59%)	54/91 (59%)	
Yes	101/247 (41%)	64/156 (41%)	37/91 (41%)	
Liver Mets				**0.003**
No	221/247 (89%)	147/156 (94%)	74/91 (81%)	
Yes	26/247 (11%)	9/156 (5.8%)	17/91 (19%)	
Bone Mets				0.175
No	132/247 (53%)	89/156 (57%)	43/91 (47%)	
Yes	115/247 (47%)	67/156 (43%)	48/91 (53%)	
Treatment Line				0.712
First Line	202/247 (82%)	126/156 (81%)	76/91 (84%)	
Second line and beyond	45/247 (18%)	30/156 (19%)	15/91 (16%)	
EGFR-TKI received				0.404
Afatinib	116/247 (47%)	72/156 (46%)	44/91 (48%)	
Erlotinib	16/247 (6.5%)	13/156 (8.3%)	3/91 (3.3%)	
Gefitinib	107/247 (43%)	67/156 (43%)	40/91 (44%)	
Osimertinib	8/247 (3.2%)	4/156 (2.6%)	4/91 (4.4%)	

Bold values represet statistical significance

*Abbreviations*: *WSE* Wood-smoke Exposure, *KPS* Karnofsky Performance Status, *ECOG PS* Eastern Cooperative Oncology Group Performance Status, *EGFR *Epidermal Growth Factor Receptor, *Mets* Metastases, *TKI* Tyrosine Kinase Inhibitor

^1^n/N (%); Median (Q1-Q3); ^2^Pearson's Chi-squared test

Laboratory and inflammatory markers differed significantly: cachectic patients had lower lymphocyte counts (median 1.10 vs 1.60; *p<*0.001) and albumin levels (median 3.50 vs 3.90; *p<*0.001), alongside higher systemic inflammation indices (NLR median 5.4 vs 3.5; *p<*0.001; PLR median 245 vs 194; *p<*0.001). Dichotomized analyses were concordant, with a higher proportion of cachectic patients meeting NLR ≥5 (54% vs 30%; *p<*0.001) and PLR ≥150 (91% vs 70%; *p<*0.001) (Table S1).

### Adverse events

**Fig. 1:**
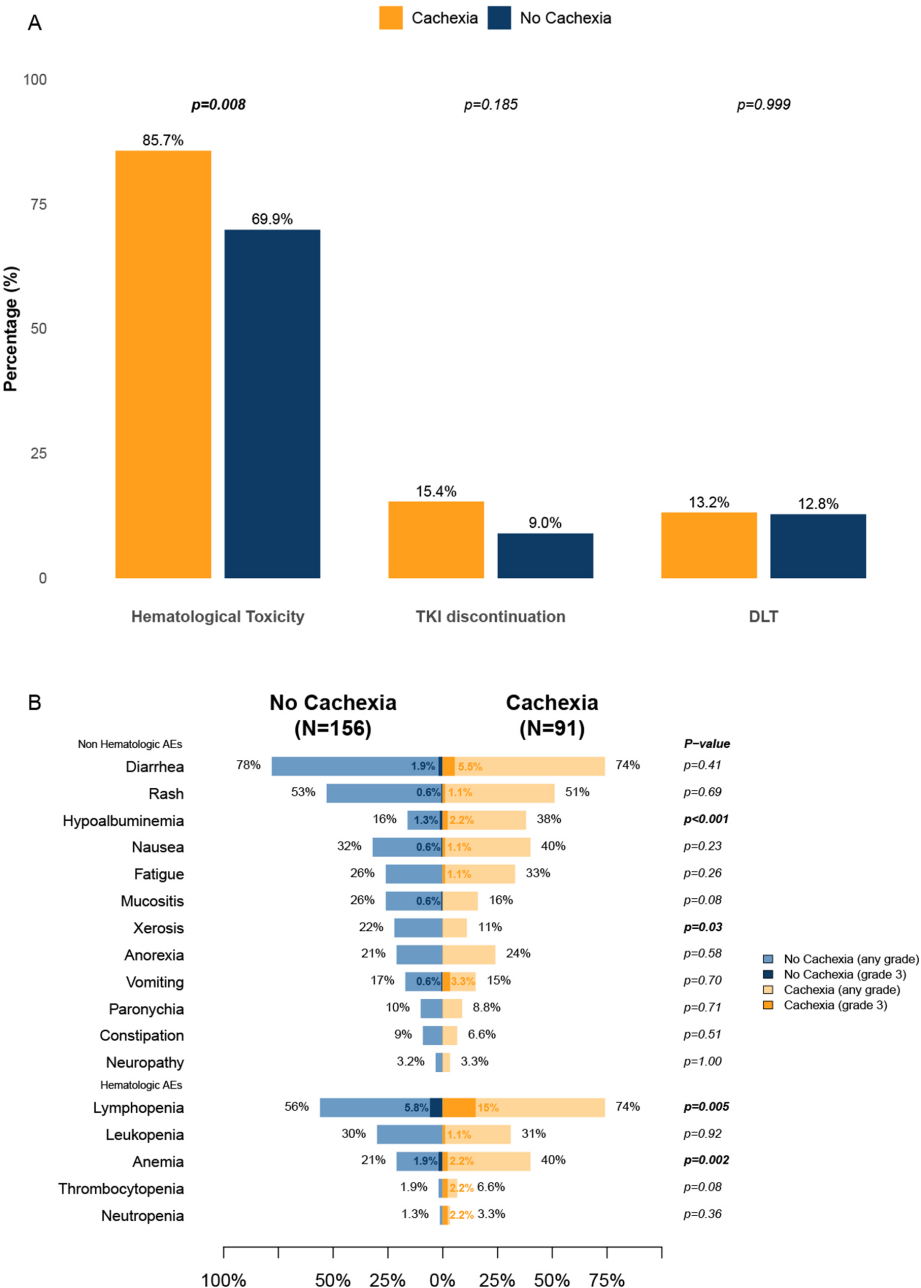
Treatment-related toxicity profile and tolerability outcomes by cachexia status. **A** Composite hematologic toxicity rate, tyrosine kinase inhibitor (TKI) discontinuation, and dose-limiting toxicity (DLT) by cachexia status, with percentages and *p*-values shown above bars. **B** Incidence of presented hematologic and non-hematologic adverse events in patients with cachexia (*N*=91) versus no cachexia (*N*=156)

Cachectic patients had a significantly higher rate of overall hematologic toxicity (85.7% vs 69.9%, *p=*0.008). By contrast, TKI discontinuation rates were comparable between groups (15.4% vs 9%, *p=*0.185), and DLT was not different (13.2% vs 12.8%, *p=*0.999) (Fig. 1A).

Overall, hematologic events were more frequent among cachectic patients, driven primarily by anemia (40% vs 21%, *p=*0.002) and lymphopenia (74% vs 56%, *p=*0.005). Grade 3 events were uncommon across categories; however, grade 3 lymphopenia appeared higher in cachectic patients (15% vs 5.8%). In contrast, most other hematologic toxicities did not differ significantly between groups (e.g., neutropenia and thrombocytopenia, both *p*>0.05). Among non-hematologic AEs, most event rates were similar between cachectic and non-cachectic patients (all *p*>0.05), including diarrhea, rash, nausea, fatigue, mucositis, anorexia, vomiting, paronychia, constipation, and neuropathy. Two notable differences were observed: hypoalbuminemia was substantially more frequent in cachectic patients (38% vs 16%, *p<*0.001), whereas xerosis was less frequent (11% vs 22%, *p=*0.03) (Fig. 1B).

In the univariate logistic regression analysis for hematologic toxicities, liver metastases showed the strongest association (OR 9.11, 95% CI 1.86–164.43; *p=*0.032), Bone metastases were also associated with increased odds (OR 2.52, 95% CI 1.37–4.78; *p=*0.0037). Cachexia remained a significant predictor (OR 2.59, 95% CI 1.34–5.28; *p=*0.0061). Regarding the multivariable model evaluating only cachexia (adjusted OR 2.26, 95% CI 1.15–4.68, *p=*0.021), and liver metastases were associated with hematological toxicity (adjusted OR 2.12, 95% CI 1.13–4.08, *p=*0.021) (Table 2).

**Table 2:** Predictive factors for Hematologic toxicity

Variable	Univariate OR (95% CI)	*p*-value^1^	Multivariate OR (95% CI)	*p*-value^1^
Sex				
Female	Reference			
Male	0.70 (0.39–1.30.39.30)	0.26		
Age (>60)				
<59	Reference			
≥60	1.25 (0.68–2.27.68.27)	0.46		
Smoking status				
Negative	Reference			
Positive	0.98 (0.54–1.82.54.82)	0.96		
EGFR type				
*EGFR*^*del19*^	Reference			
* EGFR* ^*L858R*^	1.00 (0.58–2.09.58.09)	0.78		
Uncommon	2.71 (0.47–51.05.47.05)	0.35		
ECOG PS				
≥2	Reference			
0–1	0.51 (0.16–1.29.16.29)	0.19		
Clinical Stage				
III	Reference			
IV	1.35 (0.28–5.04.28.04)	0.66		
Cachexia				
No	Reference		Reference	
Yes	2.58 (1.34–5.27.34.27)	**0.006**	2.26 (1.15–4.68.15.68)	**0.021**
Liver mets				
No	Reference		Reference	
Yes	9.10 (1.86–164.86)	**0.03**	2.11 (1.13–4.08.13.08)	**0.021**
Brain mets				
No	Reference			
Yes	1.26 (0.69–2.32.69.32)	0.44		
Bone mets				
No	Reference		Reference	
Yes	2.51 (1.36–4.77.36.77)	**0.003**	5.57 (1.08–102.08)	0.10
Sarcopenia				
No	Reference			
Yes	1.02 (0.57–1.86.57.86)	0.93		

Bold values represet statistical significance

*Abbreviations*: *OR* Odds Ratio, *CI* Confidence Interval, *ECOG PS* Eastern Cooperative Oncology Group Performance Score, *EGFR* Epidermal Growth Factor Receptor, *TKI* Tyrosine Kinase Inhibitors

^1^Binary Logistic Regression

### Survival outcomes by cachexia status

**Fig. 2:**
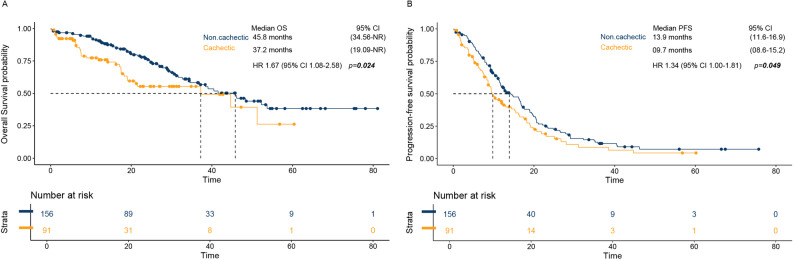
Survival outcomes by cachexia status. Kaplan–Meier curves comparing (**A**) overall survival (OS) and (**B**) progression-free survival (PFS) between cachectic and non-cachectic patients

In survival analyses, with a median follow-up of 27.1 months, OS was shorter among cachectic patients. mOS was 45.80 months in the non-cachectic group (95% CI 34.56–not reached) versus 37.22 months in the cachectic group (95% CI 19.09–not reached) (Fig. 2A).

In univariable analyses, worse OS was observed for Age ≥60 years (median OS 39.03 vs not reached for <59; HR 1.64, 95% CI 1.02–2.63; *p=*0.042). ECOG PS 0–1 was protective compared with ECOG ≥2 (median OS 45.80 vs 29.17 months; HR 0.47, 95% CI 0.27–0.82; *p=*0.008). Cachexia was associated with inferior survival (HR 1.67, 95% CI 1.08–2.58; *p=*0.021). Brain metastases were associated with shorter OS (median OS 34.56 vs 44.65 months; HR 1.57, 95% CI 1.03–2.38; *p=*0.035). EGFR mutation subtype did not significantly predict OS (*EGFR*^*del19*^ vs *EGFR*^*L858R*^: HR 1.08, 95% CI 0.68–1.71; *p=*0.753). In the multivariable model, cachexia remained an independent predictor of mortality (adjusted HR 1.65, 95% CI 1.07–2.57; *p=*0.025), together with age ≥60 years (adjusted HR 1.69, 95% CI 1.04–2.73; *p=*0.034) and brain metastases (adjusted HR 1.73, 95% CI 1.13–2.63; *p=*0.011). ECOG PS 0–1 also remained significantly protective (adjusted HR 0.53, 95% CI 0.30–0.95; *p=*0.031) (Table 3).

**Table 3:** Cox regression for overall survival

Variable	N (events)	Median OS (95% CI)	*p*-value¹	Univariate HR (95% CI)	*p*-value²	Multivariate HR (95% CI)	M *p*-value²
Sex							
Female	163 (56)	45.80 (36.76-NA)	0.278	Reference		Reference	
Male	84 (33)	34.37 (24.71-NA)		1.27 (0.82–1.95.82.95)	0.279		
Age (>60)							
<59	89 (23)	NA (32.66-NA)	**0.040**	Reference		Reference	
≥60	158 (66)	39.03 (30.62–51.22.62.22)		1.64 (1.02–2.63.02.63)	**0.042**	1.69 (1.04–2.73.04.73)	**0.034**
Smoking status							
Negative	157 (57)	40.48 (29.70-NA)	0.474	Reference		Reference	
Positive	90 (32)	46.13 (37.22-NA)		0.85 (0.55–1.32.55.32)	0.475		
EGFR type							
* EGFR* ^*del19*^	162 (59)	39.03 (31.67-NA)	0.838	Reference		Reference	
* EGFR* ^*L858R*^	76 (26)	45.80 (38.44-NA)		1.08 (0.68–1.71.68.71)	0.753		
Uncommon	9 (4)	48.33 (25.33-NA)		1.32 (0.48–3.65.48.65)	0.589		
ECOG PS							
≥2	33 (15)	29.17 (19.09-NA)	**0.007**	Reference		Reference	
0–1	214 (74)	45.80 (36.76-NA)		0.47 (0.27–0.82.27.82)	**0.008**	0.53 (0.30–0.95.30.95)	**0.031**
Clinical Stage							
III	10 (5)	39.03 (27.01-NA)	0.988	Reference		Reference	
IV	237 (84)	41.53 (34.37–53.68.37.68)		1.01 (0.41–2.48.41.48)	0.988		
Cachexia							
No	156 (56)	45.80 (34.56-NA)	**0.020**	Reference		Reference	
Yes	91 (33)	37.22 (19.09-NA)		1.67 (1.08–2.58.08.58)	**0.021**	1.65 (1.07–2.57.07.57)	**0.025**
Liver mets							
No	221 (79)	41.53 (36.76–53.68.76.68)	0.154	Reference		Reference	
Yes	26 (10)	30.62 (16.20-NA)		1.61 (0.83–3.12.83.12)	0.158		
Brain mets							
No	146 (47)	44.65 (38.44-NA)	**0.033**	Reference		Reference	
Yes	101 (42)	34.56 (22.31-NA)		1.57 (1.03–2.38.03.38)	**0.035**	1.73 (1.13–2.63.13.63)	**0.011**
Bone mets							
No	132 (45)	41.53 (32.66-NA)	0.214	Reference		Reference	
Yes	115 (44)	38.44 (25.33–53.68.33.68)		1.30 (0.86–1.97.86.97)	0.215		
Sarcopenia							
No	143 (54)	37.22 (29.70–53.68.70.68)	0.524	Reference		Reference	
Yes	104 (35)	44.65 (38.44-NA)		0.87 (0.57–1.33.57.33)	0.525		

Bold values represet statistical significance

*Abbreviations*: *OS* Overall survival, *HR* Hazard Ratio, *CI* Confidence Interval, *ECOG PS* Eastern Cooperative Oncology Group Performance Score, *EGFR* Epidermal Growth Factor Receptor, *TKI* Tyrosine Kinase Inhibitors

^1^Mantel-Cox log-rank test; ^2^Cox proportional hazards model

Similarly, PFS was inferior in cachectic patients. Median PFS was 13.90 months in non-cachectic patients (95% CI 11.66–16.92) compared with 9.79 months in cachectic patients (95% CI 8.61–15.24) (Fig. 2B). Cachexia was associated with a significantly higher risk of progression or death in the Cox model (HR 1.35, 95% CI 1.00–1.81; *p=*0.049; 192 events). Regarding the *EGFR* mutation type, *EGFR*^*del19*^ had a median PFS of 16.20 months (95% CI 12.25–17.61) versus 9.92 months (95% CI 8.15–11.96) for *EGFR*^*L858R*^, with a higher risk of progression/death (HR 1.48, 95% CI 1.09–2.01; *p=*0.013). In addition, from metastatic sites at diagnosis, patients with liver metastases had a median PFS of 12.98 months (95% CI 11.27–16.72) versus 9.46 months (95% CI 7.59–14.42) with statistically significant increased hazard (HR 2.01, 95% CI 1.27–3.18; *p=*0.003). Patients with brain metastases had a median PFS of 13.90 months (95% CI 11.43–18.23) versus 11.27 months (95% CI 9.53–15.51) without brain metastases (HR 1.50, 95% CI 1.12–2.00; *p=*0.007). Bone metastases were also associated with poorer PFS. (16.76 months; 95% CI 12.48–19.88 vs. 11.43 months; 95% CI 9.46–12.91) with increased hazard for patients with bone metastases (HR 1.53, 95% CI 1.15–2.03; *p=*0.004) (Table S2). In multivariable Cox analyses, *EGFR* subtype (*EGFR*^*L858R*^ vs *EGFR*^*del19*^) remained independently associated with shorter PFS (aHR 1.44, 95% CI 1.06–1.96; *p=*0.021). Brain metastases remained independently associated with shorter PFS (aHR 1.35, 95% CI 1.00–1.83; *p=*0.049).

## Discussion

This study aimed to investigate the impact of cachexia on toxicity and survival outcomes in patients with advanced NSCLC treated with TKIs in a real-world setting. Our findings highlight the significant role of cachexia in influencing patient outcomes and treatment-related toxicity and underscore the need for early recognition and intervention. The prevalence of cachexia in our cohort was 37%, which is consistent with previous studies [9], reinforcing the widespread occurrence of this condition in patients with advanced NSCLC and was associated with a clinically coherent phenotype: worse functional status by Karnofsky score, lower BMI with concomitant depletion of both muscle and adipose tissue on CT, and a more inflammatory laboratory profile. Clinically, cachexia was associated with a higher burden of early hematologic toxicity and with inferior overall survival, and it showed an association with shorter progression-free survival in unadjusted analyses.

It is well documented that cachexia not only negatively affects the quality of life of cancer patients, but also reduces the effectiveness of anti-cancer chemotherapy and increases its toxicity, leading to increased cancer-related mortality and medical resource expenditure [10]. However, there is limited information on this topic in the context of this study [7]. The baseline phenotype observed in cachectic patients underscores that clinically relevant nutritional risk is frequently underestimated if weight or BMI are used in isolation. Despite a median BMI still within the normal range, cachectic patients had a higher prevalence of sarcopenia and markedly lower visceral and subcutaneous adiposity, together with lower lymphocyte and albumin levels and higher NLR and PLR. The presence of liver metastases was also higher in cachectic patients, suggesting that disease distribution and/or tumor burden may contribute to or coexist with the cachexia phenotype, which aligns with its known detrimental effects on patient health and prognosis [11]. These findings underscore the need for proactive management strategies to mitigate the effects of cachexia in this patient population.

It is known that depletion of skeletal muscle leads to reduced antitumor efficacy and increased chemotherapy toxicity in NSCLC [12]. However, EGFR-TKI-based treatment has been recognized to increase response rates in patients with NSCLC [13]; nonetheless, it also promotes dermatological and gastrointestinal toxicity [14]. Gefitinib and afatinib were the most used TKIs in the present study. Grade 3 and 4 afatinib toxicities have been reported in previous studies at 7.3%, compared to 2.6% for gefitinib. Major grade 3 and 4 toxicities of afatinib include diarrhea (3%), paronychia (2.4%), and rash (1.8%) [15].

Regarding toxicity in our cohort, cachexia was associated with a higher overall incidence of hematologic adverse events during the first four months of EGFR-TKI therapy, driven mainly by anemia and lymphopenia. Grade 3 events were uncommon overall, although grade 3 lymphopenia appeared more frequent among cachectic patients. By contrast, most non-hematologic toxicities commonly attributed to EGFR-TKIs were similar by cachexia status, with the exception that hypoalbuminemia was more frequent in cachectic patients, whereas xerosis was less frequent.

Notably, our non-hematologic adverse event capture was restricted to toxicities most consistently documented in routine practice for first- and second-generation TKIs, which comprised 96% of our sample. Following our data collection plan, we only collected the presented adverse events. Consequently, other adverse events of special interest, such as pneumonitis and QT interval alterations, were not systematically assessed. These events are relatively uncommon in patients treated with afatinib or gefitinib (1%) [16], compared with osimertinib (4%) [17], and given the predominance of early-generation TKIs in our cohort, pneumonitis was not expected to be frequent.

In our study, only adverse events presented during the first four months of therapy were collected. While this approach could not characterize late-onset or cumulative toxicities that accrue over prolonged EGFR-TKI therapy, the median time-to-discontinuation due to intolerable toxicity for first and second generation TKIs is reported to be 2.56 months [18]. This aligns to the hypothesis that adverse events related to an altered metabolic state, such as cachexia, will appear in the first four months.

Importantly, despite a higher rate of hematologic toxicity, treatment discontinuation and DLTs were comparable between cachectic and non-cachectic patients. This suggests that the excess toxicity signal associated with cachexia was primarily manifested as laboratory abnormalities and milder clinical events rather than as early treatment termination in routine practice. Nonetheless, hematologic toxicities may still be clinically consequential in vulnerable patients through higher infection risk, reduced tolerance to subsequent treatments, and increased need for supportive care, reinforcing the clinical value of early risk stratification.

In regression analyses, cachexia remained associated with higher odds of hematologic toxicity, independent of bone metastases. Bone metastases also independently increased the odds of hematologic toxicity, supporting a plausible contribution of marrow involvement and reduced hematopoietic reserve. The strong univariable association observed for liver metastases showed very wide confidence intervals, consistent with small numbers and potential model instability; therefore, this signal should be interpreted as hypothesis-generating. Mechanistically, cachexia-related systemic inflammation and reduced protein-energy reserves may lower bone marrow resilience, while metastatic disease in bone and liver may further compromise hematopoiesis and drug handling.

Survival analyses support cachexia as a clinically meaningful vulnerability state in EGFR-mutated NSCLC. Cachectic patients experienced shorter median overall survival compared with non-cachectic patients. In multivariable modeling, cachexia remained independently associated with mortality, alongside age and brain metastases, while ECOG remained protective. These findings indicate that cachexia captures risk beyond performance status alone and may reflect a combined impact of inflammation, reduced physiologic reserve, and susceptibility to complications, emphasizing its important role in determining the prognosis of NSCLC patients [9, 19]. In contrast, sarcopenia did not significantly impact OS, aligning with findings from previous studies [20, 21]. Our data suggests that cachexia, a multidomain syndrome, is the dominant prognostic signal, while sarcopenia alone is insufficient once systemic inflammation and nutritional status are considered. However, studies investigating the influence of sarcopenia on OS in patients with stage III or IV NSCLC undergoing TKI treatment have yielded mixed results [7, 20, 22].

The determination of sarcopenia using the SMI measured by computed tomography at the L3 section has high accuracy and specificity, as scanning can distinguish individual tissue compositions. This method is important for diagnosing cachexia or identifying potential nutritional risk in patients with NSCLC because of its precision in discriminating and quantifying body composition, and it is considered the gold standard method for cancer patients [23]. In our study, sarcopenia was diagnosed in 55% of the cachexia group vs. 35% of the non-cachexia group, with a significantly lower SMI in the cachexia group, meeting the cut-off criteria for sarcopenia. However, it is essential to consider anthropometric parameters, as sarcopenia is not the sole determinant of nutritional status deterioration or an indicator of cachexia [24]. The present analysis clearly showed unfavorable outcomes for the cachexia group, including a significantly lower median body weight and a higher percentage of weight loss, although the BMI remained within the normal range.

For progression-free survival, cachexia was associated with shorter PFS in univariable analysis but was not retained in multivariable modeling, where EGFR subtype and brain metastases remained significant. This pattern suggests that cachexia may be intertwined with adverse disease biology and metastatic patterns that drive progression, while its strongest and most consistent contribution may be through competing risks and non-progression mortality that influence overall survival. The separation between OS and adjusted PFS signals also supports the clinical view that supportive care vulnerabilities can materially shape survival even when tumor control is primarily dictated by molecular subtype and disease sites.

These findings have practical implications for the care of patients starting EGFR-TKIs. Baseline identification of cachexia using recent weight loss, BMI, and CT-derived body composition can help flag patients at higher risk of early cytopenias and worse survival. In those patients, early referral to a multidisciplinary supportive care team (nutrition, rehabilitation/physical activity, symptom control) and closer hematologic monitoring during the initial months of treatment may be warranted. Because cachexia is potentially modifiable, systematic screening also creates a window for timely intervention before toxicities and clinical decline accumulate. This approach is consistent with international guidance that emphasizes early screening and multimodal cachexia management, individualized to disease stage and patient goals [25, 26]. Potential multimodal interventions warrant further study, including individualized nutritional support with high-protein supplementation, structured resistance exercise to preserve or rebuild lean mass, and anti-inflammatory adjuncts [25] (e.g., long-chain omega-3 fatty acids and selective COX-2 inhibition when clinically appropriate [27]), alongside emerging pharmacologic therapies that directly target cachexia biology—most notably GDF-15 blockade with ponsegromab, which improved weight gain and reduced cachexia symptoms with signals of improved activity in a randomized phase 2 trial [28].

This study has several important limitations. The retrospective, single-center design limits generalizability and lacks external validation. Although adequately powered for our primary endpoints, the sample size limited power for stratified analyses by treatment line and for detecting associations with rarer toxicity endpoints, and retrospective ascertainment of cachexia components may have introduced misclassification. Data on post-progression systemic therapies were not systematically captured, limiting our ability to assess whether treatment patterns or access to subsequent lines differed by cachexia status. Adverse events were assessed only during the first four months to capture early toxicities and standardize exposure time, but this approach cannot characterize late-onset or cumulative toxicities, and our limited scope precluded formal comparison of other important toxicities by cachexia status. Residual confounding from unmeasured variables may partly explain observed associations. Finally, cachexia was assessed only at baseline without longitudinal evaluation of body composition changes or systematic data on cachexia-directed interventions, limiting assessment of modifiable pathways. Future prospective, multicenter studies with longitudinal cachexia phenotyping, comprehensive toxicity capture, and evaluation of targeted supportive interventions are needed to validate these findings.

In conclusion, baseline cachexia identifies a subgroup of EGFR-mutated advanced NSCLC patients at increased risk for early hematologic toxicity and inferior overall survival during EGFR-TKI treatment. Incorporating systematic cachexia screening and early supportive interventions into routine care may improve treatment tolerance and outcomes in this population.This study highlights the detrimental effects of cachexia on treatment toxicity and overall survival in patients with advanced NSCLC treated with TKIs. Early recognition and intervention are crucial to mitigate these adverse effects and improve patient outcomes. Future research should focus on developing targeted interventions to manage cachexia and explore the underlying mechanisms to enhance the therapeutic efficacy of TKI treatments in this vulnerable patient population.

## Supplementary Information

Supplementary Material 1. Figure 1.

Supplementary Material 2. Table 1.

## Data Availability

The data that support the findings of this study are available on request from the corresponding author, OA. The data are not publicly available due to their containing information that could compromise the privacy of research participants.

## Abbreviations

BMI: Body mass index
CRP: C-reactive protein
CT: Computed tomography
CTCAE: Common Terminology Criteria for Adverse Events
DLT: Dose-limiting toxicity
ECOG: Eastern Cooperative Oncology Group
EGFR: Epidermal growth factor receptor
EGFR-TKI: Epidermal growth factor receptor tyrosine kinase inhibitor
HU: Hounsfield unit
IAT: Intramuscular adipose tissue
IL-6: Interleukin-6
IQR: Interquartile range
KPS: Karnofsky Performance Status
NLR: Neutrophil-to-lymphocyte ratio
NSCLC: Non-small cell lung cancer
OR: Odds ratio
OS: Overall survival
PFS: Progression-free survival
PLR: Platelet-to-lymphocyte ratio
SAT: Subcutaneous adipose tissue
SMI: Skeletal muscle index
VAT: Visceral adipose tissue
