# Supplementary figures and images for: Role of cachexia in advanced non-small cell lung cancer patients treated with EGFR-TKIS
Source: BMC Cancer. 2026 Apr 2;26:631. doi: 10.1186/s12885-026-15773-1 (PMC13188429; doi:10.1186/s12885-026-15773-1)

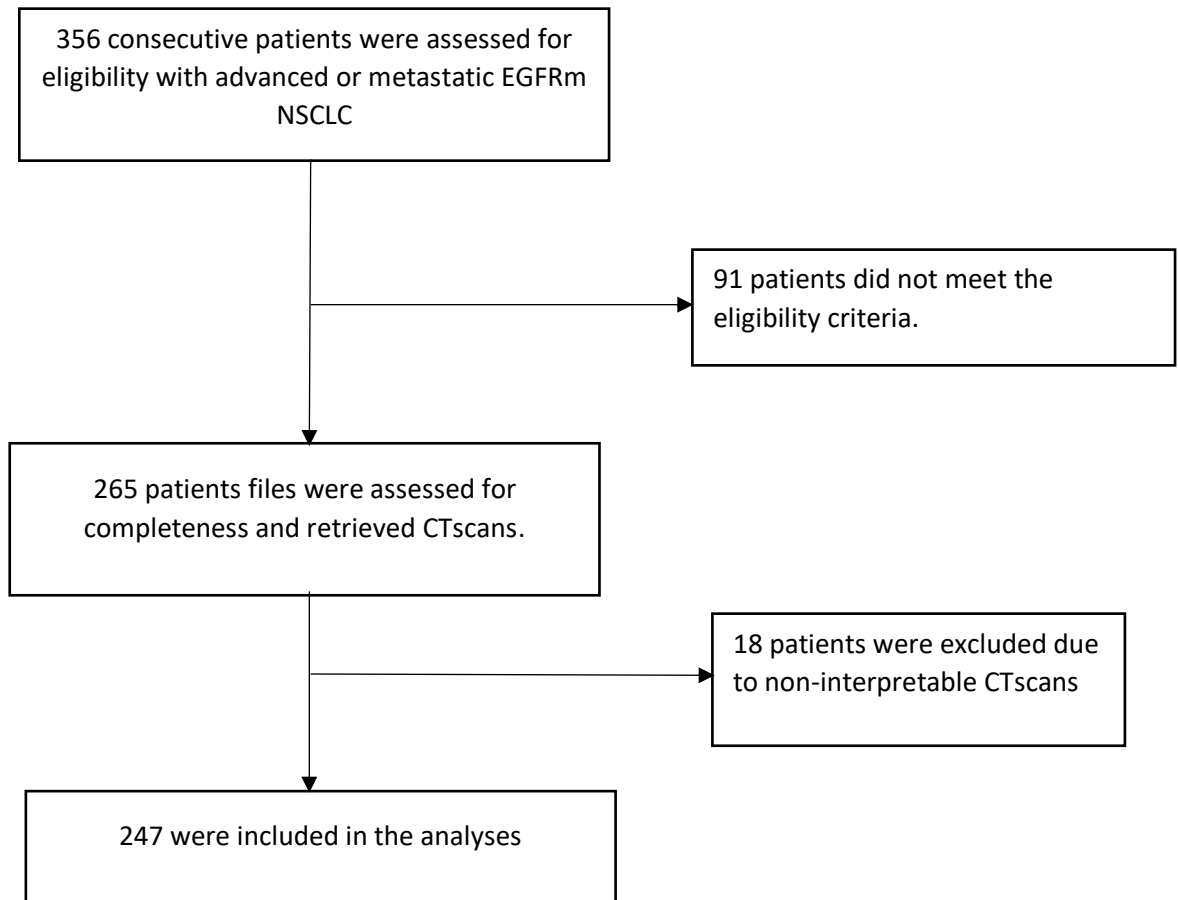

**Figure S1:** STROBE diagram.

Supplement: Supplementary file 1 — Supplementary Material 1. Figure 1. [file 12885_2026_15773_MOESM1_ESM.pdf]
