# Supplementary material for: Role of cachexia in advanced non-small cell lung cancer patients treated with EGFR-TKIS
Source: BMC Cancer. 2026 Apr 2;26:631. doi: 10.1186/s12885-026-15773-1 (PMC13188429; doi:10.1186/s12885-026-15773-1)
Supplement: Supplementary file 2 — Supplementary Material 2. Table 1. [file 12885_2026_15773_MOESM2_ESM.docx]

| ***Table S1. Antropometrical and biochemical characteristics*** | | | | |
| --- | --- | --- | --- | --- |
| **Variable** | **Overall** N = 247^1^ | **No** | **Yes** | **p-value**^2^ |
|  |  | N = 156^1^ | N = 91^1^ |  |
| **Weight (kg)** | 61 (52-70) | 62 (54-73) | 58 (48-66) | **<0.001** |
| **Height (m)** | 1.56 (1.51-1.65) | 1.56 (1.50-1.65) | 1.58 (1.52-1.65) | 0.588 |
| **BMI (kg/m2)** | 24.2 (21.4-26.8) | 25.4 (22.4-28.3) | 22.8 (19.8-25.3) | **<0.001** |
| **SMI** | 42 (36-49) | 42 (37-49) | 39 (35-48) | 0.098 |
| **SMM** | 99 (87-124) | 102 (90-127) | 98 (83-124) | 0.264 |
| **IAT** | 8 (5-14) | 8 (5-14) | 9 (6-14) | 0.626 |
| **IATI** | 3.2 (2.2-5.5) | 3.2 (2.0-5.5) | 3.4 (2.3-5.1) | 0.791 |
| **VAT** | 108 (68-159) | 120 (84-161) | 95 (48-152) | **0.006** |
| **VATI** | 45 (28-65) | 48 (34-67) | 37 (18-61) | **0.002** |
| **SAT** | 154 (100-207) | 175 (122-212) | 119 (89-179) | **<0.001** |
| **SATI** | 60 (39-85) | 70 (47-89) | 46 (34-70) | **<0.001** |
| **Hemoglobin** | 13.50 (12.40-14.70) | 13.70 (12.60-14.60) | 13.20 (11.80-14.70) | 0.116 |
| **Leucocytes** | 7.9 (5.8-9.9) | 8.1 (5.8-9.7) | 7.5 (6.0-10.0) | 0.771 |
| **Lymphocytes** | 1.30 (1.00-1.80) | 1.60 (1.10-1.90) | 1.10 (0.70-1.60) | **<0.001** |
| **Albumin** | 3.80 (3.40-4.10) | 3.90 (3.60-4.20) | 3.50 (3.10-3.90) | **<0.001** |
| **NLR** | 4.1 (2.6-6.8) | 3.5 (2.2-5.6) | 5.4 (3.0-8.9) | **<0.001** |
| **NLR dic** |  |  |  | **<0.001** |
| NLR<5 | 132 / 218 (61%) | 93 / 133 (70%) | 39 / 85 (46%) |  |
| NLR=>5 | 86 / 218 (39%) | 40 / 133 (30%) | 46 / 85 (54%) |  |
| **PLR** | 217 (164-311) | 194 (135-264) | 245 (198-384) | **<0.001** |
| **PLR dic** |  |  |  | **<0.001** |
| <150 | 48 / 217 (22%) | 40 / 132 (30%) | 8 / 85 (9.4%) |  |
| =>150 | 169 / 217 (78%) | 92 / 132 (70%) | 77 / 85 (91%) |  |
| **Platelets** | 296 (234-374) | 296 (237-368) | 296 (219-375) | 0.93 |
| ^1^n / N (%); Median (Q1-Q3) | | | | |
| ^2^Pearson's Chi-squared test; Wilcoxon rank sum test | | | | |
